# Assessing the factor structure and measurement invariance of the eating attitude test (EAT-26) across language and BMI in young Arab women

**DOI:** 10.1186/s40337-018-0199-x

**Published:** 2018-06-14

**Authors:** Salma M. Khaled, Linda Kimmel, Kien Le Trung

**Affiliations:** 1 Social and Economic Survey Research Institute, Qatar University, Doha, Qatar; 2 Institute for Social Research, University of Michigan, Ann Arbor, MI USA

**Keywords:** Disordered eating behaviors, Eating attitudes test, Measurement invariance, Body mass index, Arabic, Culture, University students, Qatar, Exploratory factor analysis, Exploratory structural equation modeling, Confirmatory factor analysis

## Abstract

**Background:**

The objective of the study was to determine the factorial structure and test the measurement invariance of the EAT-26 in a large probability sample of young female university students in Qatar (*n* = 2692), a Muslim country in the Middle East.

**Methods:**

The maximum number of factors was derived based on results from initial exploratory factor analysis (EFA) in the first-half of the randomly split sample (Sample 1). A subsequent EFA and Exploratory Structural Equation Models (ESEM) were conducted to identify the number of valid factors. A five-factor model with 19 items was identified as the optimal factor structure. This structure was further replicated using ESEM in the second-half of the sample (Sample 2). Multi-group Confirmatory Factor Analyses (CFAs) were conducted at this stage and their fit was evaluated with and without further sub-grouping by language (Arabic and English) and BMI (underweight, normal weight, and overweight/obese). Finally, measurement invariance tests were conducted in the entire sample assessing equivalence across language and BMI within the final five-factor model.

**Results:**

The five-factor structure of the new EAT-19 [fear of getting fat (FGF), eating-related control (ERC), food preoccupation (FP), vomiting-purging behavior (VPB), and social pressure to gain weight (SP)] provided the best fit: CFI = 0.976, TLI = 0.952, RMSEA = 0.045 (90%CI 0.039–0.051), SRMR = 0.018, CD =1.000. CFAs supported metric invariance for language and for BMI. Language and BMI-based population heterogeneity comparisons provided modest and small-to-moderate evidence for differential factor means, respectively.

**Conclusion:**

Although the five-factor model of the EAT-19 demonstrated good item characteristics and reliability in this young female population, the lack of scalar invariance across language and BMI-categories pose measurement challenges for use of this scale for screening purposes. Future studies should develop culture- and BMI-specific cut-offs when using the EAT as a screening instrument for disordered eating in non-clinical populations.

**Electronic supplementary material:**

The online version of this article (10.1186/s40337-018-0199-x) contains supplementary material, which is available to authorized users.

## Plain English summary

Disordered Eating Attitudes and Behaviors (DEAB) are a global phenomenon with high prevalence among young female adults in English- and non-English speaking cultures. Using exploratory and confirmatory analytical approaches in randomly half-split samples, we evaluated the theoretical structure of DEAB measured by the EAT-26 and the same structure holds across two languages (Arabic and English) and three BMI-based groups (underweight, normal weight, and overweight/obese) in a large representative sample of undergraduate female students of predominantly Arab ethnicity. A theoretical five-factor structure was supported in both samples. Although the resulting five subscales of the final EAT-19 demonstrated good internal consistency overall, other problematic measurement properties were identified for language and BMI. These properties pose serious measurement challenges for use of the EAT-26 or shorter versions for disordered eating screening purposes in young Arabic-speaking females of varying body weight. Our study highlights important implications for cross-culture research and measurement of disordered eating in non-clinical populations.

## Background

Disordered Eating Attitudes and Behaviors (DEAB) are a global phenomenon with high prevalence among young adults in English- and non-English speaking cultures. Examples of DEAB include dieting, fasting, abusing laxatives or diuretics, self-induced vomiting, and binge eating. These behaviors are associated with increased risk of eating disorders and obesity, and are a serious public health concern [1, 2]. Early identification of DEAB may be a cost-effective public health policy especially in educational settings, where the potential for intervention and follow up are feasible and inexpensive [3, 4].

In a Muslim and Arabic-speaking country like Qatar, as in many of the Gulf countries, young women constitute a high-risk population for obesity and DEAB [5–7]. Rapid urbanization and economic growth has led to high rates of obesity, a shift towards fast- and processed- foods, a sedentary lifestyle, and a greater exposure to Western ideals of thinness through the media [8, 9]. However, there are currently limited screening tools for identifying young women who are at high risk of DEAB and eating disorders in this unique cultural setting.

The Eating Attitudes Test (EAT) is one of the most widely used measures of DEAB [(10)]. Originally, 40 items (EAT-40), tested in patients with anorexia nervosa and community-based controls, it was shortened (EAT-26), psychometrically tested and validated in a mixed clinical and non-clinical English-speaking sample [10, 11]. It has since been translated and adapted to multiple languages and contexts [12].

A major challenge with the EAT-26 is its elusive factorial structure. Garner et al., (1982) proposed a three-factor model based on a principal component analysis (PCA): a dieting-factor related to avoidance of fattening foods and pre-occupation with being thinner, a bulimia- and food pre-occupation-related factor, and an oral control factor [10]. Efforts to replicate this factor structure in non-clinical populations have not been widely successful. Many studies in English-speaking countries reported four and five factors instead of three, with the number of items ranging from 16 to 25 [13–15].

In non-English speaking community samples, four to six factors have been reported [16–25]. In series of studies, Maïano and colleagues (2013) conducted a thorough investigation of the factor structure of the EAT-26 in one of the largest samples (*n* = 1779) of ethnically diverse, Europeans and Africans, populations to date. This study’s sample consisted of French-speaking, 11 to 18 years of age, adolescent boys and girls, in France [26]. Using exploratory structural equation modeling (ESEM) and confirmatory factor analysis (CFA), these authors arrived at and replicated the best fitting six-factor model with 18 items of the EAT-26. These factors included Fear of Getting Fat, Eating-Related Control, Eating Related Guilt, Food Preoccupation, Vomiting-Purging Behavior, and Social Pressure to Gain Weight.

In the Middle East, although the EAT-26 has been widely used in Arabic-speaking countries [6, 7, 27, 28], fewer studies reported on its psychometric properties [29, 30]. Nasser studied the factor structure of the Arabic version of the EAT-26 in a sample of secondary school girls in Egypt using exploratory factor analysis (EFA) to confirm the original three-factor model [29]. Although Nasser demonstrated a similar three factor-solution with 16 items, the findings were inconclusive, with high internal consistency for only one-factor, the dieting subscale [29]. Nasser concluded that the scale should only be used as a screening tool for dieting and weight-related concerns and not for bulimic behaviors [29]. Although a similar three factor structure was reported for 23 items of the EAT-26 in a recent replication in Jordan, a different pattern of item-factor loadings were reported in this study of adolescent school girls [30].

Discrepant and inconclusive factor-analytic findings have sparked debate about the factorial validity of the EAT-26 as well as its overall structure and utility as a screening tool in non-clinical samples. Differences in factor structure between English and non-English speaking countries have been largely attributed to cultural differences in eating attitudes and body-figure norms [31–33].

Another less studied, but important aspect is the demonstration of measurement invariance or equivalence across relevant subgroups i.e. the same construct is measured in every subgroup. While a few studies demonstrated measurement invariance across cultural groups for the English version of the EAT-26, this rarely has been assessed for translations [17, 24]. Demonstrating linguistic measurement invariance in mother tongue should be a prerequisite to demonstrating cultural group differences and similarities. Without it, it is not possible to rule out the possibility that observed group differences are not generalizable across languages or cultures.

Different interpretations of the EAT may also occur if body-related variables influence the meaning and interpretations of the constructs underlying the EAT. If the EAT is supposed to screen for undifferentiated eating disorder [34], then it is supposed to measure DEAB in the same way across weight status groups. If it measures different constructs across different weight categories, this may result in erroneous screening interpretations and clinical interventions based on the same EAT score. Thus, it is important to demonstrate measurement invariance across different Body Mass Index (BMI) categories. To our knowledge, measurement invariance for BMI status has not been thoroughly examined for English and other linguistic versions of the EAT. This may account for discrepancies in the factor structure of the EAT-26 across studies [35]. We found one study that assessed the measurement invariance of the EAT across BMI categories in French-speaking populations [26]. The study reported measurement invariance across underweight, normal, and overweight categories including evidence of metric and scalar invariance [26].

The main objective of this study is to re-evaluate the factor structure of the EAT-26, while examining its measurement invariance properties across two linguistic versions (English and Arabic) and BMI categories using large probability-samples of university students, predominately from Arab ethnicity. This allows us to explore sources of measurement variability that may influence the factor analytic findings and affect interpretations concerning the factorial structure of the EAT-26.

## Method

### Procedures

Translation: Since there is no standard, authorized Arabic translation, the EAT-26 was translated from English to Arabic by the first author and back translated to English by another member of the research team. Minor discrepancies in translation arose and were resolved by consensus among bilingual team members. Further conceptual validation of the Arabic translation was obtained through cognitive interviews with 20 female university students. These face to face interviews tested the students’ understanding of the EAT-26 statements; in particular their conceptual, not just literal understanding of the statements was probed and verified. Building on findings from the cognitive interviews, the questions were also piloted as part of a survey (*n* = 120) where further probing about alternative interpretations of these statements in a semi-structured manner was elicited (a list of close-ended statements based on findings from the interviews and open-ended explanations were further elicited).

Questionnaire, Survey Mode and Administration: The EAT-26 was programmed and administered in a panel study as part of a thirty-minute online questionnaire in Qualtrics [36]. Questions were included about general health, dietary habits, weight perception, and weight-related concerns and behaviors.

### Measures

Participants had a choice to complete the survey in English or Arabic, with language subgroups based on their choice. Weight status was measured using BMI (Kg/m2) based on self-reported weight and height and categorized into three groups: underweight (< 18.5), normal weight (18.5 to 24.9), and overweight or obese (25.0 or more) [37].

The EAT-26 items were measured and scored on the original six-point scale: “Never” = 1, “Rarely” = 2, “Sometimes” = 3, “Often” = 4, “Very Often” =5, and “Always” = 6. The subscales based on the factor analysis were scored as the sum of the items constituting the subscales. We did not transform the items from the original six-point scale to a four-point scale (range from 0 to 3) as per recent French study [26] and recommendation of other authors to use the original scale to preserve DEAB’s severity in non-clinical populations [34].

### Sample design

The present study is based on a two-wave panel survey of female University students. Data collection for the first wave occurred between April and May of 2016 and the second wave occurred between November 2016 and February 2017. Both waves had similar sample design and response rate (52.0 and 51.7%) [38]. A total of 3138 students completed both surveys (*n* = 1793 and *n* = 1345, respectively). After removing participants who completed both waves of the survey (*n* = 446), the remaining students constituted the total number of observations with complete responses to all EAT items, which were used in the present analysis (*n* = 2692).

Pretest and Fielding: The University’s Institutional Review Board approved an ethics compliance application for the study. Each survey data collection wave was preceded by a pre-test (*n* = 120) to check the questionnaire’s content and skip-logic and test administration logistics.

### Participants

Participants were predominantly Qataris (64.1%); the majority completed the Arabic version of the questionnaire (73.1%). Other nationalities included Egypt (5.2%), Yemen (5.0%), Palestine (4.1%), Jordan (3.8%), other Gulf countries (4.3%), other Arab countries (6.6%), Pakistan/India/Bangladesh (2.6%), Iran (2.0%), Europe/North-America (0.5%), and other Asian/Euro-Asian/African countries (1.8%). The mean age was 21.4 (standard deviation = 3.58) years (range 17–40). The mean BMI was 24.3 kg/m2 (standard deviation = 5.85) (range 11.6–72.1). The proportions of BMI categories were: 12.0% underweight, 50.9% normal weight, and 37.1% overweight or obese.

### Statistical analysis

The total number of observations from both data collection waves (*n* = 2692) were randomly split into two samples, hereafter referred to as Sample 1 and Sample 2. All analytical procedures were conducted using STATA 14 [39] to investigate the factor structure and measurement invariance of the EAT-26. Briefly, the factorial structure analysis was carried out on the first random sample (Sample 1) and was based on the six-factor EFA solution for all items of the EAT-26. We also conducted ESEM to evaluate goodness of fit for the resulting factor structure model from the EFA stage and to compare fit with other alternative models. Based on findings from Sample 1, we conducted ESEM and a series of CFA within the Structural Equation Modelling framework in the replication sample (Sample 2). The CFAs were carried out for all the observations in Sample 2 and by language and BMI. Finally, we conducted measurement invariance tests of the final factorial model on the entire sample, after combining Sample 1 and Sample 2. Below is a step-by-step account of our analytical procedures.

Factor Structure Assessment using EFA in Sample 1 (Step 1): To determine the appropriate number of factors for the 26 EAT items, EFA was conducted using principal-factor extraction, with the resulting eigenvalues (> 1) and scree plot inspected. In addition, EFA with oblique (quartimin criterion) rotation was conducted using a pre-determined number of factors from the first step to examine each item’s factor loadings and uniqueness. Loadings on each of the factors were considered low if value was less than 0.40. Uniqueness values equal or greater than 0.70 and cross-loadings equal or greater than 0.40 were also considered as evidence of poor loading.

Factor Structure Assessment using ESEM in Sample 1 (Step 2): Since the scree plot tends to overestimate the optimal number of factors, ESEM was used to assess alternative models with fewer factors [40]. ESEM is considered better suited for examining measurement properties of psychological instruments often providing more robust estimates than CFA [41, 42]. ESEM analyses with three, four, and five correlated factors were tested using the maximum likelihood robust estimator and an oblique (quartimin) rotation. Four fit indices were selected a priori to assess model fit: comparative fit index (CFI), Tucker-Lewis index (TLI), Standardized Root Mean Square (SRMS), and Root Mean Square Error of Approximation (RMSEA). Acceptable model fit was defined by a CFI ≥0.90, Tucker–Lewis index ≥0.95, SRMR or RMSEA values ≤0.08 [43, 44]. Based on these criteria, the best fitting final model was selected.

Factor Structure Assessment using ESEM and CFA in Sample 2 (Step 3): ESEM analyses with three, four, and five correlated factors were re-run in Sample 2. The final five-factor ESEM model from Sample 1 was replicated and further reexamined by CFA with and without stratification by language and BMI subgroups in Sample 2. Factor loadings, intercepts, variances, residual variances for direction of association and magnitude were inspected, and the fit statistics were evaluated using the fit indices and criteria described above.

Measurement Invariance Testing in entire sample (Step 4): Measurement invariance of the final factorial model was examined after recombining Sample 1 and Sample 2 by fitting and comparing sequentially nested and increasingly constrained CFA models across language and BMI subgroups. First, metric invariance was examined by fitting and comparing a model imposing equality in the item-factor loadings (Model 2) relative to an equal form measurement model (Model 1) [45]. This comparison tests the assumption the items have the same meaning (slopes) across subgroups [45]. To determine whether there were important differences in the item means across the different subgroups, scalar invariance (Model 3) was subsequently tested by fitting and comparing a model imposing equality in item-factor loadings and in item means (intercepts) across groups relative to a model that only imposed equality in item-factor loadings (Model 2) [45].

The tenability of invariance at each level of model constraint was determined using the following changes (∆) in fit indices criteria between a more restricted model and the preceding one: ∆CFI ≤ 0.01 or ΔRMSEA ≤0.015 or ∆TLI ≤ 0.01 [46–48]. Due to sample size-independence and correction for lack of parsimony, we considered the above criteria for ∆s in fit indices superior to ∆χ2 and the primary indicators of measurement invariance in this study [42, 46, 47].

Multiple-group Comparisons of Latent Factor Means (population heterogeneity comparisons) in Entire Sample (Step 5): While still imposing equal loadings and intercepts across subgroups, we fitted SEMs that fixed the latent factor mean for a reference group at zero then estimated the means for the other groups relative to this reference group. The equal intercepts constraint was retained to ensure that any difference in item means was reflected in the means of the latent factors [45]. Latent factor means were compared and corresponding Cohen’s d effect sizes (EFs) for their differences were estimated [49]. The EFs were considered small, moderate, and large using the following respective thresholds of 0.20, 0.50, and 0.80 [49]. EFs of value less than 0.20 were considered very small or negligible even if statistically significant at alpha of 0.05.

Correlations between the latent factors from Sample 1, Sample 2, and entire sample were assessed using Pearson product-moment correlation coefficients. To check the internal consistency in the subscales, Cronbach’s alpha coefficients were computed for Sample 1, Sample 2, and for the entire sample.

## Results

The numbering index and description in English and corresponding translation in Arabic for all the EAT-26 items appear in Additional file 1: Table S1.

**Fig. 1:**
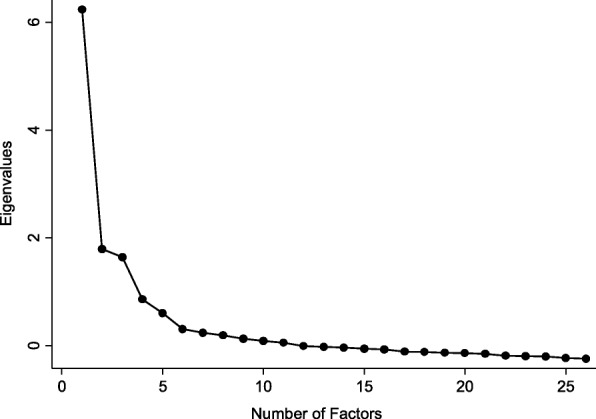
Scree plot of EAT-26 items in Sample 1

Factor Structure Assessment using EFA in Sample 1: The scree plot (Fig. 1) suggests factors beyond the first six account for little variability in the 26 items, thus the factor structure was fixed to a maximum of six factors in a second EFA (Table 1). In this analysis, seven items had unacceptably low loadings on each of the factors and/or high unique values. As a result, “I avoid eating when hungry”, “I feel a need to cut my food into small pieces”, “I take longer than others to eat my meals”, “I display self-control around food”, “I feel uncomfortable after eating sweets”, “I like my stomach to be empty”, and “I enjoy trying new nutritionally rich foods” were dropped, leaving 19 items for the subsequent analyses.

**Table 1:** Characteristics of Items, the total scale and six-factor solution based on exploratory factor analysis of the EAT-26 in Sample 1

Item #	Item Name	Mean	SD	Corrected Item-total Correlation^a^	Factor (λ)						
					1	2	3	4	5	6	δ
1	Terrified	3.43	1.83	.597	.704	−.024	.041	−.045	.062	−.061	.472
2	Hungry	2.02	1.21	.420	.194	.088	−.039	.070	.403	.067	.667
3	Preoccupy	2.68	1.38	.342	.121	−.062	.619	.021	−.053	−.050	.551
4	Binges	2.10	1.38	.441	.135	.025	.584	.034	.026	−.038	.551
5	Cutfood	2.51	1.48	.436	.231	.087	.193	−.016	.187	.126	.759
6	Awarecal	2.88	1.60	.493	.269	.371	.005	.093	−.066	−.018	.602
7	Avoidcarb	2.17	1.31	.517	.045	.498	−.045	.035	.357	−.044	.500
8	Eatmore	2.72	1.70	.023	−.053	−.106	−.018	.014	.116	.693	.517
9	Vomit	1.46	0.96	.312	−.061	.010	.017	.798	−.025	.031	.383
10	Guilty	2.59	1.60	.666	.568	−.072	.229	.118	.151	−.068	.394
11	Thinner	2.92	1.74	.692	.781	−.036	.016	.072	.059	−.023	.327
12	Burncal	3.48	1.81	.644	.689	.154	−.064	.020	−.034	−.071	.422
13	Toothin	3.03	1.77	.202	−.123	−.086	−.062	.031	−.122	.602	.563
14	Fatbody	2.69	1.65	.623	.537	−.005	.274	.030	.063	−.085	.468
15	Longeat	2.71	1.65	.131	−.014	.107	.160	.001	−.018	.236	.899
16	Avoidsug	2.33	1.34	.394	−.037	.569	−.074	.018	.182	−.011	.621
17	Dietfood	2.27	1.25	.567	.081	.669	−.012	.098	.059	−.069	.435
18	Controlf	2.63	1.56	.500	.227	.130	.440	.070	−.068	.087	.592
19	Control	3.59	1.53	.074	.165	.253	−.337	−.039	−.072	.206	.759
20	Pressure	2.55	1.49	−.016	.006	−.047	−.011	.070	.063	.684	.521
21	Muchtime	2.32	1.33	.400	.005	.103	.587	.100	−.028	.128	.563
22	Sweets	2.54	1.56	.573	.139	.249	.290	.123	.180	.008	.601
23	Dieting	2.17	1.37	.580	.012	.655	.091	.105	.107	−.169	.457
24	Stomac	2.35	1.40	.426	.188	.104	−.048	.121	.355	.129	.668
25	Vomit1	1.56	1.04	.328	.011	−.033	−.023	.811	−.041	.021	.373
26	Tryfd	3.62	1.55	.230	.027	.384	.070	−.067	−.118	.054	.834

*Note.* SD = Standard Deviation, λ = factor loading

^a^Correlation between the respective item and the EAT-26 total sum score without including the respective item

Factor Structure Assessment using ESEM in Sample 1: Three-, four- and five-factor ESEM models were run on the 19 items (Table 2). Three items had poor loadings in the three-factor model, and all fit statistics improved in the four-factor model. While the four-factor model had no items with poor loadings, all fit statistics improved in the five-factor model. In particular, the RMSEA dropped from 0.076 to 0.045, leading us to select the five-factor model for subsequent analyses.

**Table 2:** Goodness of fit indices from 3-, 4-, and 5-factor exploratory structural equation models (ESEM) of the EAT-19 in Sample 1

Descriptions	SRMR	CD	CFI	TLI	RMSEA	90% CI	χ^2^ (df)	AIC	BIC	Number of Items with Poor Loadings^b^
3 factors	.050	.994	.838	.763	.099	0.095, 0.104	1454.68 (117)^a^	72,316.85	72,781.86	3^c^
4 factors	.033	.999	.918	.860	.076	0.071, 0.081	775.69 (100)^a^	71,671.86	72,222.79	0
5 factors	.018	1.000	.976	.952	.045	0.039, 0.051	286.64 (86)^a^	71,210.81	71,832.50	1^d^

*Note. SRMR* standardized root mean square, *CD* coefficient of determination, *CFI* comparative fit index, *TLI* Tucker-Lewis index, *RMSEA* root mean square error of approximation, *CI* Confidence interval for the RMSEA point estimate, χ^2^ Chi-squared Statistic, *df* Degrees of freedom, *AIC* Akaike’s information criterion, BIC Bayesian information criterion

^a^Significant at alpha value of 0.05

^b^Factor loadings less than 0.40 is considered poor

^c^The three items with poor loadings were item 1”Terrified”,7”Vomit”,and item 19”Vomit1”

^d^The item with poor loading was item 4 “Awarecal”

In the five-factor ESEM (Table 3), no items had high cross loadings on more than one factor and the majority had loadings on a primary factor ≥ 0.60. As in EFA, similar findings were also obtained in ESEM. Furthermore, these findings were also replicated when we re-ran the ESEM in Sample 2 (not shown) with one exception. The item “I am aware of the calorie content of foods I eat” had a loading of 0.37 on factor 2 in Sample 1 (Table 3), but 0.49 on the same factor in Sample 2. Therefore, a decision was made to retain this item.

**Table 3:** Five-factor model ESEM solution for the 19-item version of the EAT in Sample 1

	ESEM					
Item	Factor (λ)					
	FGF	ERC	FP	SP	VPB	δ
Terrified	0.731	0.000	0.003	− 0.024	− 0.035	0.467
Preoccupy	0.007	−0.096	0.732	−0.087	− 0.010	0.469
Binges	0.063	0.021	0.649	−0.051	− 0.002	0.522
Awarecal	0.202	0.364	0.064	0.018	0.031	0.706
Avoidcarb	0.148	0.551	−0.050	0.030	0.011	0.593
Eatmore	0.057	−0.030	−0.013	0.780	−0.021	0.411
Vomit	−0.033	0.049	0.064	0.041	0.689	0.476
Guilty	0.634	−0.041	0.183	−0.050	0.114	0.412
Thinner	0.836	0.015	−0.021	0.021	0.049	0.287
Burncal	0.649	0.191	−0.051	− 0.035	−0.025	0.429
Toothin	−0.153	−0.048	0.010	0.597	−0.021	0.576
Fatbody	0.549	0.026	0.239	−0.053	0.015	0.475
Avoidsug	0.046	0.561	−0.114	0.041	0.002	0.640
Dietfood	−0.049	0.829	0.051	−0.010	0.004	0.338
Controlf	0.167	0.104	0.485	0.083	0.018	0.603
Pressure	0.029	0.039	0.028	0.710	0.054	0.483
Muchtime	−0.025	0.063	0.607	0.114	0.057	0.592
Dieting	−0.093	0.789	0.144	−0.116	0.024	0.375
Vomit1	0.027	−0.054	−0.090	− 0.043	1.037	0.002

*Note*. *ESEM* exploratory structural equation modeling, λ factor loading, *FGF* fear of getting fat, *ERC* eating-related control, *FP* food preoccupation, *SP* social pressure to gain weight, *VPB* vomiting-purging behavior, δ uniqueness

The five factors obtained from ESEM in Sample 1 (Table 3), which was also replicated using ESEM in Sample 2 (not shown) represent the constructs of Fear-of-Getting-Fat, Eating-Related-Control, Food-Preoccupation, Vomiting-Purging-Behavior, and Social-Pressure-to-Gain-Weight. These factors explained 47.0% of the variation in the 19 items. Acceptable fit statistics were obtained for the final five-factor ESEM model (CFI = 0.976, TLI = 0.952, RMSEA = 0.045). With the exception of the correlations between Fear-of -Getting-Fat and Eating-Related-Control (*r* = 0.662) and Fear-of-Getting-Fat and Food-Preoccupation (*r* = 0.571), most factors had small or minimal correlations (seven of the remaining eight *r* ≤ 0.35) (Table 4). Similar correlations between latent factors were also obtained in Sample 2 (not shown) and in the entire sample (not shown).

**Table 4:** Correlations between Latent Factors in Sample 1

	EAT- Factor				
Correlations	FGF	ERC	FP	SP	VPB
FGF	1.00				
ERC	.662*	1.00			
FP	.571*	.268*	1.00		
SP	−.234*	−.050	−.066*	1.00	
VPB	.276*	.256*	.349*	.181*	1.00

*p < 0.01 for the latent factor correlation

*FGF* fear of getting fat, *ERC* eating- related control, *FP* food preoccupation, *SP* social pressure to gain weight, *VPB* vomiting-purging behavior

Factor Structure Assessment using CFA in Sample 2: The five-factor CFAs had acceptable fit statistics for the full Sample 2 (CFI = 0.913, TLI = 0.895; RMSEA = 0.067) with all 19 items having loadings ≥0.40 (Table 5). Acceptable fit statistics were obtained for language-based CFAs for English (CFI = 0.900, TLI = 0.879; RMSEA = 0.074) and Arabic (CFI = 0.907, TLI = 0.887; RMSEA = 0.070). Similarly, acceptable fit statistics were found for underweight (CFI = 0.851, TLI = 0.821; RMSEA = 0.075); normal weight (CFI = 0.928, TLI = 0.913, RMSEA = 0.056); and overweight or obese (CFI = 0.858, TLI = 0.829; RMSEA = 0.079).

**Table 5:** Goodness-of-fit for the EAT-19 5-factor CFI models for entire sample and across different groups of language and BMI categories in Sample 2

	Descriptions	χ^2^ (df)	CFI	TLI	RMSEA	SRMR	CDC
CFA in Sample 2							
(*N* = 1112)	19 items, 5-factor solution^a^	854.624 (142)	0.913	0.895	0.067	0.060	0.999
CFA by Language							
Arabic (*n* = 867)	19 items, 5-factor solution^a^	742.618 (141)	0.907	0.887	0.070	0.063	0.999
English (*n* = 245)	19 items, 5-factor solution^a^	334.104 (142)	0.900	0.879	0.074	0.066	0.999
CFA by BMI							
Underweight (*n* = 122)	19 items, 5-factor solution^a^	238.954 (142)	0.851	0.821	0.075	0.076	0.998
Normal (*n* = 517)	19 items, 5-factor solution^a^	375.021 (142)	0.928	0.913	0.056	0.050	0.999
Overweight or Obese (*n* = 346)	19 items, 5-factor solution^b^	447.522(142)	0.858	0.829	0.079	0.075	1.000

*Note*. *CFA* confirmatory factor analysis, *CFI* comparative fit index, *TLI* Tucker-Lewis index, *CI* confidence interval, *RMSEA* Root Mean Square Error of Approximation; Ref model is the reference model for the measurement invariance comparison; χ^2^ = Chi-squared Statistic, df = degrees of freedom

^a^All items had loadings ≥0.40

^b^All items had loadings ≥0.40 except item “Other people think I am too thin”

Measurement Invariance in the Entire Sample: The results supported metric invariance only for language based on the change criteria in fit statistics specified a priori (Table 6): Model 2 vs. Model 1 [∆CFI = − 0.001, ∆TLI = + 0.003, and ∆RMSEA = − 0.001]. In contrast, scalar invariance was not supported for language and failed to meet the allowed change statistics with a decrease exceeding 0.01 for ∆CFI: Model 3 vs. Model 2 [∆CFI = − 0.014, ∆TLI = − 0.009, and ∆RMSEA = + 0.003]. The tests for measurement invariance based on three BMI categories supported metric invariance only (Table 6): Models 2 vs. Model 1 [∆CFI = − 0.009, ∆TLI = − 0.002, and ∆RMSEA = 0.000]. In contrast, scalar invariance was not supported for BMI-based categories (Table 6) and failed to meet the allowed change range in fit statistics with a decrease exceeding 0.01 for both CFI and TLI, while the increase in RMSEA exceeding 0.015: Model 3 vs. Model 2 [∆CFI = − 0.114, ∆TLI = − 0.111, and ∆RMSEA = + 0.026].

**Table 6:** Measurement invariance for the EAT-19 5-factor model by language and BMI categories in combined sample (Sample 1 and Sample 2)

	Descriptions	χ^2^ (df)	CFI	TLI	RMSEA	Ref Model	Δχ^2^(df)	∆ CFI	∆TLI	∆RMSEA
Multi-group Analysis by Language (*N* = 2270)										
Model 1	Equal Form (Unrestricted)	1741.53 (284)	0.912	0.895	0.067	–	–	–	–	–
Model 2	Equal loadings (Metric)	1780.09 (298)	0.911	0.898	0.066	1	38.56 (14)	−.001	+.003	−.001
Model 3	Equal loadings & intercepts (Scalar Invariance)	2033.66 (317)	0.897	0.889	0.069	2	253.57(19)	−.014	−.009	+.003
Multi-group Analysis by BMI (*N* = 2048)										
Model 1	Equal Form (Unrestricted)	1606.04 (426)	0.905	0.885	0.064	–	–	–	–	–
Model 2	Equal loadings (Metric)	1737.03 (454)	0.896	0.883	0.064	1	131.00(28)	− 0.009	−0.002	0.000
Model 3	Equal loadings & Intercepts (Scalar Invariance)	3196.09 (492)	0.782	0.772	0.090	2	1459.06(38)	−0.114	− 0.111	+ 0.026

*Note*. *CFI* comparative fit index, *TLI* Tucker-Lewis index, *CI* confidence interval, *RMSEA* Root Mean Square Error of Approximation; Ref model is the reference model for the measurement invariance comparison; χ^2^ = Chi-squared Statistic, df = degrees of freedom

Multiple-group Comparisons of Latent Factor Means (population heterogeneity comparisons) in the Entire Sample: For the language comparison, the differences in latent factor means are presented in Table 7. Although four out of the five factors (exception factor Social-Pressure-to-Gain-Weight) demonstrated statistically significant mean differences, the corresponding EFs were considered negligible for all five factors. For the BMI comparisons, all factors except Vomiting-Purging Behavior had statistically significant mean differences and small to moderate EFs especially across the overweight and obese BMI category relative to the underweight reference category.

**Table 7:** Multiple-group comparisons of latent factor means and their corresponding effect sizes in combined sample

	FGF					ERC					FP					SP					VBP				
	*∆ Latent Mean*	*Pooled SD*	*Effect Size*	*P-Value*	*95% CI*	*∆ Latent Mean*	*Pooled SD*	*Effect Size*	*P-Value*	*95% CI*	*∆ Latent Mean*	*Pooled SD*	*Effect Size*	*P-Value*	*95% CI*	*∆ Latent Mean*	*Pooled SD*	*Effect Size*	*P-Value*	*95% CI*	*∆ Latent Mean*	*Pooled SD*	*Effect Size*	*P-Value*	*95% CI*
Language* * (N = 2270)*																									
*English* * (n = 521)*	−.155	2.28	−.068	.021	−0.29, 0.02	0.225	1.72	.130	.000	0.13, 0.32	.156	1.77	.088	.003	0.05, 0.26	−.103	2.54	−.040	.169	−0.25, 0.04	.158	1.51	.105	.000	0.07, 0.25
BMI** (*N = 2048)*																									
*Normal Weight (n = 1052)*	.888	2.61	.340	.000	0.74, 1.04	.444	1.92	.231	.000	0.33, 0.55	.224	2.22	.101	.001	0.10, 0.35	−1.33	3.29	−.406	.000	−1.52, −1.14	−.056	2.06	−.027	.357	−0.17, 0.06
*Overweight/Obese (n = 747)*	2.01	3.04	.663	.000	1.84, 2.19	0.967	2.192	0.441	.000	0.84, 1.09	0.752	2.587	0.291	.000	0.60, 0.90	−2.323	3.490	−0.666	.000	−2.52, −2.12	0.042	2.205	0.019	.513	−0.08, 0.17

*Notes: SD* Standard Deviation, *CI* Confidence Interval, *FGF* fear of getting fat, *ERC* eating- related control, *FP* food preoccupation, *SP* social pressure to gain weight, *VPB* vomiting-purging behavior, *BMI* Body Mass Index

*Baseline for language base comparison is Arabic (*n* = 1749)

**Baseline for BMI comparison was underweight BMI category (*n* = 249)

Descriptive statistics and Cronbach alphas of the 19-item five-factor subscales of the original EAT-26 are shown for the entire sample and by language and BMI sub-groups (see Table 8). The internal consistency was reasonably good for all subscales (ranging from 0.725 to 0.845). The mean scores for all subscales except Vomiting-Purging-Behavior were generally lower for respondents who completed the questionnaire in English than Arabic. Those who reported being overweight or obese had generally higher mean scores across Fear-of-Getting-Fat, Eating-Related-Control, and Food-Preoccupation subscales than those in the normal and underweight categories. For Social-Pressure-to-Gain-Weight, the opposite pattern of decreasing mean score with increasing BMI was observed, while for Vomiting-Purging-Behavior, all three BMI categories had similar mean scores (see Table 8).

**Table 8:** Means, standard deviations and Cronbach’s alpha for 5-factor EAT-19 subscales in the total sample and across languages and BMI status in combined sample

	EAT Subscales														
	FGF			ERC			FP			SP			VPB		
	*M* (SD)	*95% CI*	*α*	*M* (SD)	*95% CI*	*α*	*M* (SD)	*95% CI*	*α*	*M* (SD)	*95% CI*	*α*	*M* (SD)	*95% CI*	*α*
Total Sample (*N* = 2692)	9.88 (6.64)	9.64, 10.12	.845	9.91 (6.35)	9.68, 10.14	.806	8.13 (5.11)	7.13, 8.31	.731	6.98 (4.71)	6.81, 7.15	.725	2.48 (1.92)	2.41, 2.55	.809
Arabic (*N* = 1968)	10.48 (6.57)	10.21, 10.76	.853	10.17 (5.99)	9.92, 10.43	.793	8.34 (4.89)	8.14, 8.55	.733	7.36 (4.59)	7.16, 7.55	.720	2.53 (1.84)	2.45, 2.60	.818
English (*N* = 724)	8.22 (6.55)	7.76, 8.68	.818	9.20 (7.21)	8.70, 9.70	.841	7.55 (5.63)	7.16, 7.94	.724	5.95 (4.87)	5.61, 6.29	.738	2.34 (2.11)	2.19, 2.49	.774
Underweight (*N* = 320)	6.93 (3.91)	6.47, 7.40	.766	8.5 (4.08)	8.01, 8.99	.743	7.95 (3.88)	7.49, 8.41	.711	12.32 (4.03)	11.85, 12.80	.653	2.86 (1.73)	2.65, 3.06	.762
Normal Weight (*N* = 1373)	10.14 (5.20)	9.84, 10.44	.812	10.79 (5.20)	10.49, 11.09	.801	8.82 (3.78)	8.60, 9.04	.659	8.69 (3.86)	8.47, 8.91	.684	2.76 (1.64)	2.67, 2.84	.800
Overweight or Obese (*N* = 999)	14.55 (5.52)	14.18, 14.92	.790	13.27 (5.49)	12.90, 13.64	.769	10.71 (4.81)	10.38, 11.03	.771	6.02 (3.08)	5.81, 6.23	.603	2.95 (1.92)	2.82, 3.08	.818

*Note. M* mean, *SD* Standard Deviation, *CI* Confidence Interval, α Cronbach’s alpha

*FGF* fear of getting fat, *ERC* eating- related control, *FP* food preoccupation, *SP* social pressure to gain weight, *VPB* vomiting-purging behavior

## Discussion

The main aim of this study was to examine the factor structure of the EAT-26 in a non-clinical probability sample of young females of predominantly Arab ethnicity. The optimal factor structure was derived based on results from EFA and ESEM in the first-half of the randomly split sample. This structure was further replicated in the second-half of the sample and its fit was evaluated with and without further sub-grouping by language- and BMI. Additionally, measurement invariance tests were conducted in the entire sample assessing equivalence across language and BMI. Successive multi-group comparisons using SEM within the final five-factor CFA model tested for metric and scalar invariance and for population heterogeneity through comparison of latent factor means across these groups [50].

The resulting five-factor structure was similar to the six-factor structure reported in one of the largest factorial validation studies conducted to date on this topic using ethnically diverse French adolescents by Maïano et al. (2013) with four of the five factors comprising the same items [26]. The main observed difference in the factor structure (six versus five factors) between the abovementioned study and ours was caused by three items: “I feel extremely guilty after eating”, “I feel uncomfortable after eating sweets”, and “I like my stomach to be empty”. While, all these items loaded satisfactorily on a sixth factor, Eating-Related-Guilt factor in the other study, this was not the case for these items in our study. In particular, the item “I feel extremely guilty after eating” had significant loading on the Fear-of-Getting-Fat factor, while the other two items had no loadings above 0.40 on any factor in our study. Thus, the latter two items were dropped from our analysis at the EFA stage along with other poor-loading items. Maïano et al. (2013) eliminated the same other items that we also dropped from our study (“I avoid eating when hungry”, “I feel a need to cut my food into small pieces”, “I take longer than others to eat my meals”, “I display self-control around food”, and “I enjoy trying new nutritionally rich foods”) with the exception of “I cut my food into small pieces”. Poor loadings were also reported for all these items in the first replication of Garner’s findings in a sample of Arab girls in Egypt [29].

Despite some overlap, our results differed from the three-factor solutions proposed by two previous studies of the Arabic EAT-26 [29, 30]. Two of our factors, Fear-of-Getting Fat and Eating-Related-Control, were in the dieting factor in Nasser’s study [29] and dieting and awareness of food content in the second study [30]. The emergence of Fear-of-Getting-Fat as a unique factor in our study is consistent with the theoretical notion that distortion in body shape perception is a construct central to eating-related psychopathology and is distinct from eating-related restriction behaviors (as measured by Eating-Related-Control) [51, 52]. In this regard, our results are also consistent with CFA findings from a recent study treating perception of body shape as a separate latent factor from dieting [15].

Another important finding we share with Maïano and colleagues [26], is the support of Vomiting-Purging-Behavior as a unique factor independent of Food-Preoccupation or binge eating tendencies. While Vomiting-Purging-Behavior is an independent latent factor in our study, both “I vomit after I have eaten” and “I have impulse to vomit after meals” had poor loadings (− 0.01 and 0.12) in Nasser’s study [29]. In a more recent replication of that study, the latter was dropped, while the former item was retained within the Food-Preoccupation factor [30]. Future studies should investigate the relation between these two factors and what determines their co-occurrence (as in Bulimia Nervosa) or independence.

Our results supported weak factorial invariance in the measurement of DEAB across the two languages, a minimum requirement to meet before carrying out other theoretically important between-group comparisons [50]. However, evidence emerged against equal items’ means (intercepts), thus we failed to provide support for scalar invariance in the Arabic and English versions of the 19-item EAT.

Similar to our findings with respect to language, evidence for metric invariance was observed across BMI groups, supporting equal meaning ascribed to the same items by underweight, normal weight, overweight, and obese participants. However, our results did not support scalar invariance of the EAT-19 leading to the conclusion of weak factorial invariance of the EAT-19 for BMI-based categories [35]. This finding is inconsistent with reported strict invariance of an 18-item French version of the EAT-26 [26].

When further inspecting latent factor means for population heterogeneity, we found that the EFs for the differences in all the latent means for English versus Arabic were modest with negligible values. However, unlike language, we found that when comparing overweight and obese categories versus underweight category, the latent factors’ means, except for Vomiting-Purging-Behavior factor, were substantially different indicating the levels of the latent factors vary across groups. Specifically, individuals who are overweight and obese scored significantly higher than individuals who are underweight on Fear-of-Getting-Fat, Eating-Related-Control, and Food-Preoccupation, but significantly lower on Social-Pressure-to-Gain-Weight. This could be due to several reasons including variability in the magnitude of correlations between the latent factors across BMI categories, other psychometric properties of the EAT, and the self-report nature of our assessment method. Alternatively, it is also possible that the latent constructs that the EAT taps, as well as the five-factor model tested here, may differ across BMI categories. Future studies should clarify the present findings especially in light of previous findings indicating that the EAT can be used as a screening tool in non-clinical populations for undifferentiated eating disorders [34].

### Limitations, strengths, and future directions

One major limitation of this study is that we could not establish invariance of the EAT-19 using second-order sub-grouping, such as measurement equivalence between Arabic and English within the four BMI-based categories due to sample size limitation. Future studies should endeavor to replicate or disconfirm our findings using these finer sub-groupings. A second limitation is our reliance on self-reported weight and height for BMI. The web-based administration of the EAT could reduce the generalizability of our findings to interviewer-administered questionnaires. While our sample had a good representation of female students from all over the Arab world, it is unclear whether our findings would generalize to males, females of different ages, or Arab females of lower educational status.

## Conclusion

Our findings supported the five-factor solution for 19 EAT items with largely satisfactory consistency values for the resulting five subscales. Additionally, we found evidence of weak invariance across BMI-based categories as well as Arabic and English versions of the EAT-19. However, our study found a lack of scalar invariance across both language and BMI-categories, posing challenges for use of this scale for screening purposes in young Arab females. This finding is problematic for clinical screening purposes because it would mean that even when levels on the DEAB construct are identical, young Arab females belonging to different BMI-groups would still score higher or lower on the different items, giving the false impression of higher or lower levels of DEAB. Further, research into measurement invariance by BMI and cultural groups of the EAT are needed.

Specifically, the current threshold values for delineating potential cases for clinical follow-up should be adapted to reflect ethnicity and BMI-based status. In addition, separate population-based norms for the EAT score should be established for different BMI-categories and for Arabic-speaking populations. In light of our findings and the current established utility of the EAT in screening for DEAB, we recommend future studies to develop culture- and BMI-specific cut-offs when using the EAT as a screening instrument for DEAB and risk of eating disorder.

## Additional file

Additional file 1: **Table S1.** Eating attitude test (EAT) – Items’ numbering index and description. (DOCX 22 kb)

## Abbreviations

BMI: Body Mass Index
CFA: Confirmatory Factor Analysis
DEAB: Disordered Eating Attitudes and Behaviors
EAT: Eating Attitudes Test
EFA: Exploratory Factor Analysis
ESEM: Exploratory Structural Equation Modeling

## References

[1] Fairburn CG, Welch SL, Doll HA, Davies BA, O’Connor ME (1997). Risk factors for bulimia nervosa. A community-based case-control study. Arch Gen Psychiatry.

[2] Fairburn CG, Doll HA, Welch SL, Hay PJ, Davies BA, O’Connor ME (1998). Risk factors for binge eating disorder: a community-based, case-control study. Arch Gen Psychiatry.

[3] Austin S, Ziyadeh N, Forman S, Prokp L, Keliher A, Jacobs D. Screening high school students for eating disorders: results of a national initiative. Prev Chronic Dis. 2008;5(4):1–10. PMC2578782 18793502

[4] Mond JM, Hay PJ, Rodgers B, Owen C (2007). Health service utilization for eating disorders: findings from a community-based study. Int J Eat Disord..

[5] Musaiger AO (2011). Overweight and obesity in eastern Mediterranean region: prevalence and possible causes. J Obes.

[6] Musaiger AO, Al-Mannai M, Tayyem R, Al-Lalla O, Ali EYH, Kalam F (2012). Prevalence of overweight and obesity among adolescents in seven Arab countries: a cross-cultural study. J Obes.

[7] Musaiger AO, Al-Mannai M, Tayyem R, Al-Lalla O, Ali EYA, Kalam F (2013). Risk of disordered eating attitudes among adolescents in seven Arab countries by gender and obesity: a cross-cultural study. Appetite.

[8] Madanat HN, Brown RB, Hawks SR (2007). The impact of body mass index and western advertising and media on eating style, body image and nutrition transition among Jordanian women. Public Health Nutr.

[9] Sreedharan J, Antony A, Qureshi S, Fazal S, Siddiqui H, et al. Media Influence on the Body Image Among Students in UAE. J Community Med Health Educ. 2012;2:182.

[10] Garner DM, Olmsted MP, Bohr Y, Garfinkel PE (1982). The eating attitudes test: psychometric features and clinical correlates. Psychol Med.

[11] Garner DM, Garfinkel PE (1979). The eating attitudes test: an index of the symptoms of anorexia nervosa. Psychol Med.

[12] Nasser M (1997). The EAT speaks many languages: review of the use of the EAT in eating disorders research. Eat Weight Disord EWD.

[13] Doninger GL, Enders CK, Burnett KF (2005). Validity Evidence for Eating Attitudes Test Scores in a Sample of Female College Athletes. Meas Phys Educ Exerc Sci.

[14] Mumford DB, Whitehouse AM, Platts M (1991). Sociocultural correlates of eating disorders among Asian schoolgirls in Bradford. Br J Psychiatry J Ment Sci.

[15] Ocker LB, ETC L, Jensen BE, Zhang JJ (2007). Psychometric Properties of the Eating Attitudes Test. Meas Phys Educ Exerc Sci.

[16] Ambrosi-Randić N, Pokrajac-Bulian A (2005). Psychometric properties of the eating attitudes test and children’s eating attitudes test in Croatia. Eat Weight Disord EWD..

[17] Belon KE, Smith JE, Bryan AD, Lash DN, Winn JL, Gianini LM (2011). Measurement invariance of the eating attitudes Test-26 in Caucasian and Hispanic women. Eat Behav.

[18] Choudry IY, Mumford DB (1992). A pilot study of eating disorders in Mirpur (Pakistan) using an Urdu version of the eating attitudes test. Int J Eat Disord.

[19] Douka A, Grammatopoulou E, Skordilis E, Koutsouki D (2009). Factor analysis and cut-off score of the 26-item eating attitudes test in a Greek sample. Biol Exerc.

[20] Koslowsky M, Scheinberg Z, Bleich A, Mark M, Apter A, Danon Y (1992). The factor structure and criterion validity of the short form of the eating attitudes test. J Pers Assess.

[21] Lee AM, Lee S (1996). Disordered eating and its psychosocial correlates among Chinese adolescent females in Hong Kong. Int J Eat Disord..

[22] Leichner P, Steiger H, Puentes-Neuman G, Perreault M, Gottheil N (1994). Validation of an eating attitude scale in a French-speaking Quebec population. Can J Psychiatry Rev Can Psychiatr.

[23] Nasser M (1994). Screening for abnormal eating attitudes in a population of Egyptian secondary school girls. Soc Psychiatry Psychiatr Epidemiol.

[24] Rutt CD, Coleman KJ (2001). The evaluation of a measurement model for the body image questionnaire and the eating attitudes test in a Hispanic population. Hisp J Behav Sci.

[25] Szabo CP, Allwood CW (2004). A cross-cultural study of eating attitudes in adolescent South African females. World Psychiatry.

[26] Maiano C, Morin AJS, Lanfranchi M-C, Therme P (2013). The eating attitudes Test-26 revisited using exploratory structural equation modeling. J Abnorm Child Psychol.

[27] Al-Adawi S, Dorvlo ASS, Burke DT, Moosa S, Al-Bahlani S (2002). A survey of anorexia nervosa using the Arabic version of the EAT-26 and “gold standard” interviews among Omani adolescents. Eat Weight Disord - Stud Anorex Bulim Obes.

[28] Thomas J, Khan S, Abdulrahman AA (2010). Eating attitudes and body image concerns among female university students in the United Arab Emirates. Appetite.

[29] Nasser M (1994). The psychometric properties of the eating attitude test in a non-western population. Soc Psychiatry Psychiatr Epidemiol.

[30] Mousa T, Beretvas SN (2016). Factor structure of scores of an Arabic version of the eating attitude test. J Hum Nutr Food Sci.

[31] Ball K, Kenardy J (2002). Body weight, body image, and eating behaviours: relationships with ethnicity and acculturation in a community sample of young Australian women. Eat Behav.

[32] Nasser M (1988). Culture and weight consciousness. J Psychosom Res.

[33] Nasser M (1988). Eating disorders: the cultural dimension. Soc Psychiatry Psychiatr Epidemiol.

[34] Mintz LB, O’Halloran MS (2000). The eating attitudes test: validation with DSM-IV eating disorder criteria. J Pers Assess.

[35] Meredith W (1993). Measurement invariance, factor analysis and factorial invariance. Psychometrika.

[36] Qualtrics software, Version 37,89237,892 of the Qualtrics Research Suite. Copyright © 2016 Qualtrics. Qualtrics, Provo, UT, USA. https://www.qualtrics.com.

[37] WHO | Obesity: preventing and managing the global epidemic [Internet]. WHO. 2000 [cited 23 Apr 2017]. Available from: http://www.who.int/entity/nutrition/publications/obesity/WHO_TRS_894/en/index.html

[38] Standard Definitions: Final Dispositions of Case Codes and Outcome Rates for Surveys. The American Association for Public Opinion Research; 2016. Report No.: 9th Edition.

[39] StataCorp. Stata Statistical Software: Release 13 [Internet]. College Station, TX, USA: StataCorp LP; 2013. (Stata Statistical Software: Release 13). Available from: http://www.stata.com/support/faqs/resources/citing-software-documentation-faqs/.

[40] Velicer WF, Eaton CA, Fava JL, Goffin RD, Helmes E (2000). Construct explication through factor or component analysis: a review and evaluation of alternative procedures for determining the number of factors or components. Problems and solutions in human assessment.

[41] Asparouhov T, Muthén B (2009). Exploratory Structural Equation Modeling. Struct Equ Model Multidiscip J.

[42] Marsh HW, Muthén B, Asparouhov T, Lüdtke O, Robitzsch A, Morin AJS (2009). Exploratory structural equation modeling, integrating CFA and EFA: application to students’ evaluations of university teaching. Struct Equ Model Multidiscip J.

[43] Bentler PM (1990). Comparative fit indexes in structural models. Psychol Bull.

[44] Hu L, Bentler PM (1999). Cutoff criteria for fit indexes in covariance structure analysis: conventional criteria versus new alternatives. Struct Equ Model Multidiscip J.

[45] Acock A (2013). Chapter 5 group comparisons: 5.3.4Testing for equal intercepts. Discovering structural equation modeling using Stata, revised edition.

[46] Chen FF (2007). Sensitivity of goodness of fit indexes to lack of measurement invariance. Struct Equ Model Multidiscip J.

[47] Cheung GW, Rensvold RB (2002). Evaluating goodness-of-fit indexes for testing measurement invariance. Struct Equ Model Multidiscip J.

[48] Marsh H, Hau K-T, Grayson D (2005). Goodness of fit evaluation in structural equation modeling. Contemporary psychometrics a Festschrift for Roderick P McDonald.

[49] Cohen J (1988). Statistical power analysis for the behavioral sciences Second.

[50] Bryant F, Yarnold P (1995). Principal-Components Analysis and Exploratory and Confirmatory Factor Analysis. Reading and Understanding Multivariate Statistics 1st ed.

[51] American Psychiatric Association. Diagnostic and Statistical Manual of Mental Disorders. 2013 [cited 12 Apr 2017]. Available from: http://dsm.psychiatryonline.org/doi/book/10.1176/appi.books.9780890425596

[52] Thompson JK, Altabe M, Johnson S, Stormer SM (1994). Factor analysis of multiple measures of body image disturbance: are we all measuring the same construct?. Int J Eat Disord.

