# Supplementary material for: Assessing the factor structure and measurement invariance of the eating attitude test (EAT-26) across language and BMI in young Arab women
Source: J Eat Disord. 2018 Jun 14;6:14. doi: 10.1186/s40337-018-0199-x (PMC6001056; doi:10.1186/s40337-018-0199-x)
Supplement: Supplementary file 1 — Table S1. Eating attitude test (EAT) – Items’ numbering index and description. (DOCX 22 kb) [file 40337_2018_199_MOESM1_ESM.docx]

| **Additional file 1: Table S1: Eating attitude test (EAT) – Items’ numbering index and description** | | | | |
| --- | --- | --- | --- | --- |
| EAT-26 Item | EAT-40 Item | Item Name | English Item Description | Arabic Item Description |
| 1 | 4 | Terrified | I am terrified about being overweight | أنا أرتعب من أن يكون وزني زائداً |
| 2 | 5 | Hungry | I avoid eating when I am hungry | أتجنب تناول الطعام حين أكون جائعة |
| 3 | 6 | Preoccupy | I find myself preoccupied with food | أجد نفسي منشغلة بالطعام |
| 4 | 7 | Binges | I have gone eating binges where I feel I may not be able to stop | سبق أن مررت بنوبات من الإفراط في الأكل حيث أشعر أنني عاجزة عن التوقف |
| 5 | 8 | Cutfood | I cut my food into small pieces | أشعر بالحاجة إلى تقطيع طعامي إلى أجزاء صغيرة |
| 6 | 9 | Awarecal | I aware of the calorie content of foods I eat | نا على علم بما تحتويه الأطعمة التي آكلها من سعرات حرارية |
| 7 | 10 | Avoidcarb | I particularly avoid food with a high carbohydrate content (bread, rice, potatoes, etc.) | أتجنب الأطعمة الغنية بالنشويات خصوصاً (مثل الخبز والأرز والبطاطا...) |
| 8 | 12 | Eatmore | I feel that others would prefer if I ate more | أشعر بأن الآخرين يفضلون لو أنني آكلت أكثر |
| 9 | 13 | Vomit | I vomit after I have eaten | أنا أتقيأ بعد أن أتناول الطعام |
| 10 | 14 | Guilty | I feel extremely guilty after eating | أشعر بذنب شديد بعد الاكل |
| 11 | 15 | Thinner | I am preoccupied with a desire to be thinner | أنا منشغلة برغبتي في أن أكون نحيفة |
| 12 | 22 | Burncal | I think about burning up calories when I exercise | أفكر في حرق السعرات الحرارية حين أمارس الرياضة البدنية |
| 13 | 24 | Toothin | Other people think I’m too thin | يظن الآخرون أنني نحيفة جداً |
| 14 | 25 | Fatbody | I am preoccupied with the thought of having fat on my body | أنا منهمكة في التفكير في تكدس السمنة بجسمي |
| 15 | 26 | Longeat | I take longer than others to eat my meals | أستغرق وقتاً أطول من غيري لتناول وجباتي |
| 16 | 29 | Avoidsug | I avoid foods with sugar in them | أتجنب الأطعمة التي تحتوي على السكر |
| 17 | 30 | Dietfood | I eat diet foods | أتناول الأطعمة الخاصة بالحمية الغذائية |
| 18 | 31 | Controlf | I feel that food controls my life | أشعر بأن الطعام يتحكم بحياتي وبسلوكي |
| 19 | 32 | Control | I display self-control around food | استطيع أن أتحكم بالطعام الذي أتناوله |
| 20 | 33 | Pressure | I feel other pressure me to eat more | أشعر بأن الآخرين يضغطون عليّ لكي آكل |
| 21 | 34 | Muchtime | I give too much time and thought to food | أكرس نسبة كبيرة من الوقت والتفكير للطعام |
| 22 | 36 | Sweets | I feel uncomfortable after eating sweets | أشعر بعدم الارتياح بعد تناول الحلويات |
| 23 | 37 | Dieting | I engage in dieting behavior | أتبع حمية غذائية معينة |
| 24 | 38 | Stomac | I like my stomach to be empty | أحب أن تكون معدتي فارغة |
| 25 | 40 | Vomit1 | I have impulse to vomit after meals | لديّ نزعة إلى التقيؤ بعد تناول الوجبات |
| 26 | 39 | Tryfd | I enjoy trying new rich foods | أستمتع بتجربة الأطعمة الغنية بالعناصر المغذية |
